# Level of Dental Anxiety and Its Role Among Barriers to Habitual Use of Oral Health Care in Adult Finns

**DOI:** 10.3390/dj14050306

**Published:** 2026-05-18

**Authors:** Vesa Pohjola, Anna Liisa Suominen, Mika Kajita, Pirjo Kurki, Ulla Harjunmaa, Satu Lahti

**Affiliations:** 1 Department of Community Dentistry, University of Turku, 20014 Turku, Finland; mika.ogawa@utu.fi (M.K.); satu.lahti@utu.fi (S.L.); 2 Research Unit of Population Health, Faculty of Medicine, University of Oulu, 90014 Oulu, Finland; 3 Institute of Dentistry, University of Eastern Finland, 70211 Kuopio, Finland; liisa.suominen@uef.fi (A.L.S.) pirjo.kurki@uef.fi (P.K.); 4 Oral and Maxillofacial Teaching Unit, Primary Oral Health Care Services, Wellbeing Services County of North-Savo, 70211 Kuopio, Finland; 5 Welfare Epidemiology and Monitoring Unit, Finnish National Institute for Health and Welfare, 00271 Helsinki, Finland; 6 Unit of Services, Department of Healthcare and Social Welfare, Finnish Institute for Health and Welfare, 00271 Helsinki, Finland; ulla.harjunmaa@thl.fi; 7 Centre for Population Health Research, University of Turku, 20520 Turku, Finland; 8 Turku University Hospital, 20520 Turku, Finland

**Keywords:** habitual use of oral health care, dental fear, dental anxiety, cost, queues, transport connections, perceived treatment need, adults

## Abstract

**Objectives**: The aims were to compare prevalence of high dental anxiety (DA) in 2000, 2011 and 2023, to compare the age- and sex-specific levels of DA, and to study if total, anticipatory, and treatment-related DA have an independent association with the non-habitual use of oral health care considering age, sex, education, current perceived treatment need, and reported barriers to use of oral health in nationally representative samples of adult Finns (n = 1950). **Methods**: DA was assessed with a single question (n = 1770) for prevalence and with the Modified Dental Anxiety Scale (MDAS) (n = 1739) for DA levels described as means, medians, standard errors (SE), interquartile ranges, and 95% confidence intervals (CI). Multivariable logistic regression analyses were used to assess the independent effect of total, anticipatory, and treatment-related DA on habitual use of oral health care (regular = habitual, for toothache or other problems = non-habitual) adjusted for age, sex, level of attained education, current perceived treatment need, and barriers to using oral health care (care costs and long queues). **Results**: Among women, the prevalence of high DA decreased from 2000 to 2011, but the decrease did not continue between 2011 and 2023. Among men, the prevalence of high DA decreased between 2000 and 2023. The mean MDAS (SE) for women was 10.1 (0.1) and for men 8.4 (0.1). Total, anticipatory, and treatment-related dental DA had an independent association with non-habitual use of oral health services. **Conclusions**: DA as an independent barrier to oral health care can prevent habitual care utilization, potentially leading to poor oral health.

## 1. Introduction

Dental fear and anxiety, later referred to as dental anxiety (DA), are very common phenomena in populations. According to a meta-analysis including 31 studies, the global estimated prevalence of high DA among adults was 11.2% [1]. In Finland, about 10% of adults have reported high DA, and about 30% moderate DA [2]. Among adult Finns aged 30+ years, the prevalence of high DA decreased in an 11-year follow-up [2]. A decreasing trend in DA was also observed among Australian adults in a 12-year follow-up [3].

The most severe consequence of DA is irregular or symptomatic use or avoidance of oral health care [4,5,6,7,8,9,10,11,12], which can have adverse consequences and lead to deterioration of oral health [13,14,15,16]. This has been described as a vicious cycle of dental fear [4,17,18]. Determinants for utilization of oral health care are predisposing factors (demographic characteristics and social structure, i.e., age, gender, education, and DA), enabling factors (assisting people to use services, i.e., accessibility, cost, and income), and need factors (i.e., oral health and treatment need) [19,20,21]. In addition to DA, the high financial cost of care has been identified in several populations as the main reason for not seeking oral health care, even among those with treatment needs [22,23,24,25,26,27,28,29]. In Finland, a considerable proportion of adults have reported cost and long queues as barriers to accessing oral health care services [30], and those with unmet treatment needs have reported a poor economic situation and high DA [31,32]. Also, a lack of perceived treatment need has often been listed as a barrier to habitual use of oral health care [29,33].

Many factors associated with non-habitual use of oral health care have also been associated with DA. Women report DA more often than men and people with lower education more often than people with higher education [1,5,6,11,12,34]. Young people have reported DA more often than older people, but the results are inconsistent [1,5,6,11]. People with high DA are more likely to have poor perceived or professionally determined oral health and to need oral health treatment more often than people with no or low DA [1,5,17,29,34].

In DA, two constructs have been distinguished: anticipatory dental DA, with avoidance aspects, and treatment-related DA, with stimulus-specific fear [35,36]. Anticipatory DA reflects future-oriented cognitive apprehension (i.e., anticipation and waiting), and treatment-related DA reflects in-procedure, somatic/conditioned fear of invasive stimuli (drilling, injection) [36]. Anticipatory DA and treatment-related DA have been distinguished, i.e., in the Dental Fear Survey and Modified Dental Anxiety Scale (MDAS) [35,36]. The two constructs of MDAS have been found in large studies conducted in different populations [36,37].

Nationally representative epidemiological studies on the relationship between DA and oral health service use are rare [3,4,5,6,11,12]. Additionally, many large studies on DA and oral health care have covered only specific groups. In addition, most studies have not simultaneously included DA and several other barriers to habitual use of oral health care [22,23,24,25,26,27,28,38,39,40]. Therefore, our aims were (1) to compare nationally representative age- and sex-specific prevalence of high DA, obtained from a single question, in 2023 to changes in earlier national studies in 2000 and 2011, (2) to compare the age- and sex-specific mean levels of DA measured with MDAS in 2023, and (3) to study if DA and its two constructs, anticipatory and treatment-related DA, have an independent association with the non-habitual use of oral health care when considering age, sex, education, current perceived treatment need, and reported barriers to use of oral health in 2023.

## 2. Materials and Methods

### 2.1. Study Design and Participants

The nationally representative Healthy Finland Survey targeted the adult population aged 20 years and older. The data were gathered in 2022–2023 using stratified, two-stage cluster sampling, questionnaires, and health examinations. The study, including the sample design and the number of individuals during different phases, was described in detail in the Cohort Profile: Healthy Finland Survey [41]. The study followed ethical standards (i.e., the Declaration of Helsinki), ethical principles for medical research involving human subjects, and the European Code of Conduct for Research Integrity. The Institutional Review Board of the Finnish Institute for Health and Welfare conducted an ethical review of the questionnaire survey (THL/72/6.02.01/2022). For the health examination, the Helsinki and Uusimaa Hospital District Regional Committee on Medical Research Ethics conducted the ethical review (decision number HUS/900/2022). Written informed consent was obtained from each individual participating in the health examination [41]. For the health examination, a sub-sample was selected (n = 9973), and one-third of the participants were invited to a clinical oral health examination (n = 3482) [42]. The participants were from the 15 largest cities on the mainland of Finland. Additionally, participants from eight randomly selected health centers from the university hospital districts were invited [42]. To obtain representative results for the target population (urban and rural), the Healthy Finland survey used two-stage stratified cluster sampling and weight coefficients in the analyses. Weights were produced using the Inverse Probability Weighting method, including data available for the entire sample (age, sex, marital status, socioeconomic group, occupation, native language, the urbanity of the area of residence, health status, and health service use) [41,42].

During the health examination, those invited to the clinical oral health examination received an oral health-related questionnaire that asked about, i.e., habitual use of oral health services, barriers to use of oral health care, current treatment need, and DA. The questionnaire was answered by 1950 participants (56%) [42]. Compared to non-participants, the study participants were older, more highly educated, and predominantly female and native Finnish speakers. For more details, see Suominen et al. (2025) [42]. The questionnaires used in the study are available from the Finnish Institute for Health and Welfare [43].

### 2.2. Measures

#### 2.2.1. Dental Anxiety

DA was assessed with two measures, a single question and the Modified Dental Anxiety Scale (MDAS). The single question, “Do you find visiting the dentist a frightening experience?” with answer options ‘Not at all’, ‘Yes, somewhat frightening’, and ‘Yes, very frightening’ was chosen to assess the prevalence of DA because of its validity and reliability for measuring DA in Finnish populations [44]. In addition, the same question was used in the national Health 2000 and 2011 surveys [45,46,47] and answered by 8081 and 4091 participants, respectively. Details of the national Health 2000 and 2011 surveys can be found at https://urn.fi/URN:NBN:fi:thl:ak:169e7a10-3cb3-472c-8413-0d0dbb2cfc90 and https://urn.fi/URN:NBN:fi:thl:ak:b331061b-9282-4d1d-8674-0675423ad51f (accessed on 13 May 2026), and the details for the reported DA for those aged 30+ years in Liinavuori et al. (2016) [2]. In this study, the prevalence of DA was reported for those aged 20+ years (1770 responders), and those reporting being very frightened were categorized as having high DA, as in 2000 and 2011.

The mean level of DA was measured using the MDAS, which is valid and reliable [33,48,49,50], and is also available in Finnish [51]. In the MDAS, DA is measured with five questions: (1) “If you went to your dentist for treatment tomorrow, how would you feel?” (2) “If you were sitting in the waiting room (waiting for treatment), how would you feel?” (3) “If you were about to have a tooth drilled, how would you feel?” (4) “If you were about to have your teeth scaled and polished, how would you feel?” and (5) “If you were about to have a local anesthetic injection in your gum, above an upper back tooth, how would you feel?” There are five response alternatives ranging from 1 (not anxious) to 5 (extremely anxious). Sum scores for MDAS (all items, range = 5–25) and two subscales, anticipatory DA (items 1 and 2; score range = 2−10) and treatment-related DA (items 3, 4, and 5; score range = 3−15), were calculated [36,50]. All five MDAS questions were answered by 1739 participants, the anticipatory DA questions by 1769 participants, and the treatment-related DA questions by 1743 participants.

#### 2.2.2. Habitual Use and Barriers to Oral Health Care

The habitual use of oral health care services was assessed with the question: ‘Do you visit oral health care?’ with reply alternatives ‘Regularly, i.e., according to the individual recall intervals’ (forming category ‘Habitual’), ‘Only when you have a toothache or some other problem’, and ’Never’ (both included in the category ‘Non-habitual’). The last two reply options were combined because the number of those who answered ‘Never’ was very low. This categorization also followed the categorization used in the previous national studies in Finland and makes the comparison with the previous results easier. Answers to this question were received from 1783 participants.

Barriers to using oral health care were assessed with the question: ‘Have any of the following factors prevented you from getting oral health care services?’ Response options were Yes/No for the following items: ‘Long queues for oral health care’, ‘Poor transport connections to the care facility’, ‘Excessive service fees and prices’, ’Dental anxiety’, ’Unprofessional conduct in the care facility’, or ‘Some other reason’. A total of 1752 participants answered this question. The current perceived need for oral health care services was assessed with the question: ‘Do you think you are currently in need of oral health care?’ (Yes/No) and answered by 1736 participants.

#### 2.2.3. Sociodemographic Factors

This study included age, sex, and educational level as sociodemographic characteristics, and these data were obtained from national registers. The age was determined in years and categorized into 20–29, 30–39, 40–49, 50–59, 60–69, and 70+ years. Sex was categorized as men or women. The classification of education was based on UNESCO’s International Standard Classification of Education 2011 (https://uis.unesco.org/sites/default/files/documents/isced-2011-en.pdf, accessed on 15 May 2026), and the information for educational level was retrieved from Statistics Finland (https://stat.fi/en/luokitukset/koulutusaste/koulutusaste_1_20160101, accessed on 15 May 2026). The educational level was categorized into three categories: low (early childhood education, primary education, lower secondary education), medium (upper secondary education, post-secondary non-tertiary education), or high (short-cycle tertiary education, bachelor’s or equivalent level, master’s or equivalent level, doctoral or equivalent level).

### 2.3. Statistical Analysis

The data analysis was performed using SAS Callable SUDAAN software (Release 11.0) to consider the two-stage cluster sampling design and weight coefficients. Inverse probability weights were used in the analyses to adjust for differences in sample selection and participation probabilities, reducing potential bias due to non-participation. Descriptive statistics for DA were calculated as percentages, means, medians, standard errors (SE), interquartile ranges, and 95% confidence intervals (CI). Sthe statistical significance of the bivariate association between habitual use of oral health care and DA, age, sex, level of education, current perceived treatment need, and reported barriers to using oral health care (long queues to oral health care, poor transport connections, excessive service fees and prices, DA and unprofessional conduct in the care facility) was analyzed by chi-square tests for categorical and Wald F-test for discrete variables, and by logistic regression analyses, with the habitual use of oral health care being the dependent variable, and DA the independent variable. Multivariable logistic regression analyses were used to assess the independent effect of DA on the habitual use of oral health care. Three separate models were used. Model 1 included the total sum of MDAS (MDAS questions 1–5), Model 2 included the sum of anticipatory DA (MDAS questions 1 and 2), and Model 3 included the sum of treatment-related DA (MDAS questions 3–5). Separate models were used to avoid multicollinearity. Spearman correlation coefficients were used for MDAS total and MDAS anticipatory DA (0.86), and for MDAS total and MDAS treatment-related DA (0.97).

The multivariable models were adjusted for age, sex, level of education, current treatment need, and barriers to using oral health care (costs and long queues). Poor transport connections to the care facility and unprofessional conduct in the care facility were not included in the models because the participants very seldom reported them as barriers to habitual use of oral health care. As the one-point difference in the MDAS score is not very clinically meaningful, we also calculated ORs for 3-, 5-, and 10-point differences in MDAS total and 3- and 5-point differences in anticipatory and treatment-related MDAS scores.

## 3. Results

**Figure 1:**
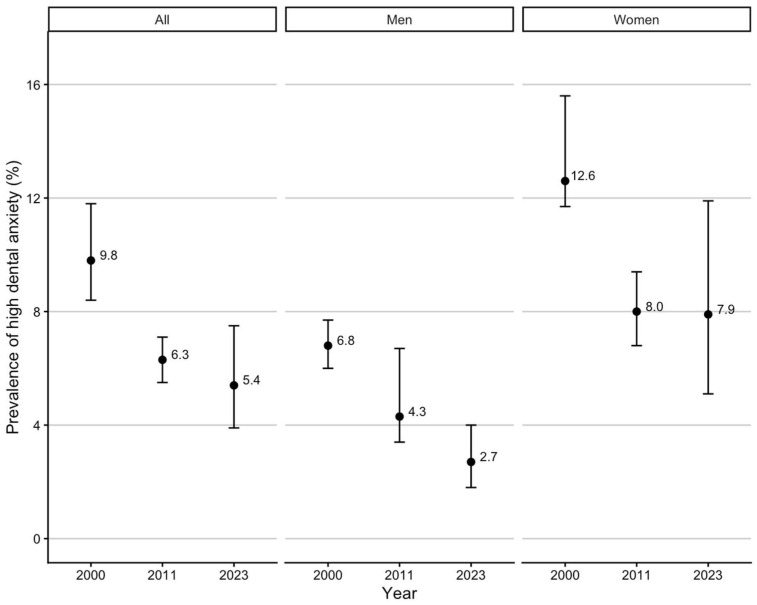
Prevalence (%) and 95% CI of high DA from a single question in 2000, 2011, and 2023 by sex. The data from the national surveys of 2000 and 2011 form a follow-up survey for those aged 30+ in 2000. The data for 2023 is from a separate cross-sectional sample.

The comparison of high DA in the Finnish national surveys from 2000, 2011, and 2023 is illustrated by sex for the total population in Figure 1. Among women, the prevalence of high DA decreased from 2000 to 2011, but the decrease did not continue between 2011 and 2023. Among men, the prevalence of high DA decreased between 2000 and 2023. The comparison of high DA in the Finnish national studies by age group and sex is shown in Table 1. The observed decrease between 2000 and 2011 across several age groups and sexes did not clearly continue between 2011 and 2023.

Generally, women reported higher levels of total DA as well as anticipatory and treatment-related DA. Younger age groups tended to report higher overall DA, anticipatory, and treatment-related DA, but only the oldest age group differed from other age groups (Table 2). The highest mean and median MDAS values were reported by 30–39-year-olds.

More than half of the population used oral health care services non-habitually (Table 3). All mean MDAS values were higher among non-habitual than habitual users of services. Non-habitual users were more often men than women, belonged to younger age-groups, and often had low or medium education. Non-habitual users reported all barriers to use of oral health care more often than habitual users. Poor transport and unprofessional conduct were very seldom reported as barriers to the use of oral health care. Non-habitual users reported less often current treatment need than habitual users. Of those with high DA, 68.9% reported DA as a barrier to using oral health care (data not presented in the tables).

**Table 3:** Description of study population by habitual and non-habitual use of oral health care services according to sociodemographic, socioeconomic factors, dental anxiety (DA), factors preventing oral health care, and current perceived treatment need.

		All		Habitual		Non-Habitual		
		n	%	n	%	n	%	*p*-Values
Use of oral health care		1783	100	998	48.8	785	51.2	
								
Dental anxiety		n	Mean (SE)	n	Mean (SE)	n	Mean (SE)	
MDAS total		1739	9.3 (0.1)	973	8.8 (0.1)	748	9.8 (0.2)	<0.001 *
MDAS, Anticipatory		1769	3.3 (0.04)	984	3.1 (0.1)	767	3.5 (0.1)	<0.001 *
MDAS, Treatment-related		1743	6.0 (0.1)	975	5.8 (0.1)	750	6.3 (0.1)	0.003 *
Sociodemographic and socioeconomic factors		n	%	n	%	n	%	
Sex (n = 1783)	Men	769	47.5	372	39.3	397	56.1	<0.001
	Women	1014	52.5	626	60.7	388	43.9	
Age group, years (n = 1783)	20–29	218	14.3	80	9.2	138	19.6	0.004
	30–39	321	18.0	152	15.4	169	20.8	
	40–49	269	15.3	143	15.2	126	15.5	
	50–59	312	16.4	193	18.8	119	13.9	
	60–69	320	16.0	215	19.6	105	12.2	
	70+	343	20.0	215	21.9	128	17.9	
Education (n = 1783)	Low	227	18.1	105	14.3	122	22.1	<0.001
	Medium	706	45.0	335	39.7	371	50.5	
	High	850	36.9	558	46.0	292	27.4	
Barriers to using oral health care (N = 1752)								
Long queues for oral health care	No	1208	68.6	735	73.2	473	63.7	<0.001
	Yes	544	31.4	247	26.8	297	36.3	
Poor transport connections to the care facility	No	1729	98.2	974	99.0	755	97.4	0.015
	Yes	23	1.8	8	1.0	15	2.6	
Costs, excessive service fees/prices	No	1469	83.4	880	89.6	589	77.0	<0.001
	Yes	283	16.6	102	10.4	181	23.0	
DA as a barrier	No	1577	89.7	932	94.1	645	85.0	<0.001
	Yes	175	10.3	50	5.9	125	15.0	
Unprofessional conduct in the care facility	No	1698	96.8	964	97.6	734	96.0	0.106
	Yes	54	3.2	18	2.4	36	4.0	
Current perceived treatment need	No	1009	59.6	452	46.3	557	73.5	<0.001
	Yes	727	40.4	521	53.7	206	26.5	

SE = standard error; MDAS = Modified Dental Anxiety Scale; *p*-values for chi-squared test, except those marked with * for Wald F-test.

In the logistic regression analyses, an independent association of total, anticipatory, and treatment-related DA with non-habitual use of oral health services was found. Table 4 presents the OR for a difference of one MDAS point. Adjustment only modestly attenuated the bivariate associations. For the MDAS total sum, the ORs (CI 95%) for non-habitual use of services for differences of three, five, and ten points in MDAS were 1.1 (1.03–1.2), 1.2 (1.1–1.4), and 1.5 (1.1–2.0), respectively. The ORs (CI 95%) for non-habitual use of services for differences of three and five points in anticipatory DA were 1.3 (1.0–1.7) and 1.6 (1.0–2.5), and in treatment-related DA were 1.2 (1.0–1.3) and 1.3 (1.0–1.6), respectively.

**Table 4:** Summary of findings of the bivariate unadjusted and adjusted (Models I–III) logistic regression analyses on the association between dental anxiety and non-habitual use of oral health care among adults in Finland in 2023.

		Unadjusted	Adjusted		
			Model I (n = 1681)	Model II (n = 1710)	Model III (n = 1684)
		OR (95% CI)			
Intercept			0.31 (0.15–0.61)	0.28 (0.15–0.55)	0.33 (0.17–0.64)
Dental Anxiety					
MDAS ^1^		1.06 (1.03–1.09) ^2^	1.04 (1.01–1.07) ^2^		
MDAS Anticipatory ^1^		1.18 (1.09–1.27) ^2^		1.12 (1.03–1.22) ^2^	
MDAS Treatment-related ^1^		1.07 (1.02–1.12) ^2^			1.05 (1.00–1.10) ^2^
Age		0.98 (0.97–0.99)	0.98 (0.97–0.99)	0.98 (0.97–1.00)	0.98 (0.97–0.99)
Sex	Men	1.97 (1.62–2.41)	2.17 (1.81–2.61)	2.17 (1.81–2.60)	2.16 (1.79–2.61)
	Women (ref.)	1.0	1.0	1.0	1.0
Educational level	Low	2.61 (1.87–3.64)	2.90 (1.78–4.72)	3.10 (1.96–4.9)	2.91 (1.79–4.72)
	Medium	2.13 (1.75–2.60)	1.85 (1.37–2.48)	1.81 (1.34–2.44)	1.87 (1.40–2.49)
	High (ref.)	1.0	1.0	1.0	1.0
Barriers to oral health care					
Costs	Yes	2.58 (2.00–3.37)	2.11 (1.64–2.70)	2.02 (1.57–2.60)	2.12 (1.65–2.72)
	No (ref.)	1.0	1.0	1.0	1.0
Queues	Yes	1.56 (1.22–1.98)	1.19 (0.83–1.72)	1.17 (0.80–1.69)	1.20 (0.83–1.75)
	No (ref.)	1.0	1.0	1.0	1.0
Current perceived treatment need	Yes (ref.)	1.0	1.0	1.0	1.0
	No	3.21 (2.71–3.82)	2.92 (2.36–3.60)	2.88 (2.33–3.57)	2.92 (2.36–3.60)

^1^ Discrete variable; ^2^ OR for the difference of one MDAS point. Outcome coding: non-habitual use of oral health care = 1, habitual use = 0; MDAS = Modified Dental Anxiety Scale; OR = odds ratio; CI = confidence interval.

## 4. Discussion

In Finnish national studies, the prevalence of high DA decreased between 2000 and 2011, but the decreasing trend did not clearly continue between 2011 and 2023. Among women, the prevalence of high DA decreased from 2000 to 2011 but did not continue between 2011 and 2023. Among men, the prevalence of high DA decreased between 2000 and 2023. Younger age groups and women reported higher levels of DA measured by MDAS than older age groups and men. The highest DA was reported by 30–39-year-olds. DA overall, as well as anticipatory and treatment-related DA, acted as independent barriers to habitual use of oral health care.

The decline in the prevalence of high DA observed in Finnish national studies among women between 2000 and 2011 and among men between 2000 and 2023 is in concordance with the limited evidence reported for adult populations [3,7,10,39,40]. A decreasing trend in DA was found in the longitudinal component of Australian National Studies of Adult Oral Health between 2004–2006 and 2017–2018 [3]. In two cross-sectional Swedish national studies, a decreasing trend in DA was seen between 1962 and 2013 [10]. Similarly, a decreasing trend in DA was observed in the cross-sectional studies conducted in 1996 and 2016 among 18-year-olds [40] and in 1997 and 2007 among 25-year-olds in Norway [7]. However, in a longitudinal cohort study in New Zealand that included adolescents, an increasing trend in DA was detected between ages 15 and 26, followed by a decreasing trend between 26 and 32 years of age [39]. This is partly in concordance with the attenuating decreasing trend between 2011 and 2023, where the direction of changes according to point-estimates varied across age groups. These findings from longitudinal studies suggest that changes in the prevalence of DA may be due to an age effect rather than a cohort effect.

The age at which the highest prevalence of high DA has been observed has varied across national cross-sectional studies, depending on the DA measures used, the age groups included, the period during which the studies were conducted, and the countries. The highest prevalence of high DA in this study was found among 40–49-year-olds, in the national UK survey in 2009 among 25–34-year-olds [12], in a national New Zealand study among 35–54-year-olds [11], and in a national Australian study among 46–64-year-olds [4].

Women reported higher mean levels of DA than men, which corresponds with previous findings. In this study, the mean MDAS for women (10.1) and for men (8.4) were lower than that in the 2009 national survey in the United Kingdom (11.8 for women and 9.3 for men) [12] but higher than in a Norwegian study involving over 20,000 participants aged 40 or older (8.3 for women and 7.1 for men) [16]. The difference in mean MDAS between the national UK study in 2009 and this study was 0.9 for men and 1.7 for women, but the UK study [12] was conducted 14 years earlier. In the Norwegian study, the mean MDAS figures were lower, but the participants were also older. In this study, younger age groups tended to report higher levels of DA, which is in accordance with previous findings. The highest MDAS mean values were reported in this study by 30–39-year-olds, in the national UK 2009 survey by 16–34-year-olds [12], and in the Norwegian study by 40–49-year-olds, which was the youngest age group in that study [16].

DA remained associated with non-habitual use of oral health care even after adjustment for other commonly reported barriers and sociodemographic factors, suggesting that DA may represent a distinct psychological barrier rather than merely reflecting cost-related or structural barriers. High cost was also a barrier to habitual use of oral health care services in this study. These findings align with previous studies, which identified DA and high cost as primary reasons for non-habitual use of oral health care services [22,23,24,25,26,27,28,29]. Notably, anticipatory DA showed a stronger association with non-habitual use than treatment-related DA. This may indicate that anxiety arising before the dental visit, such as worry about making or attending an appointment, is more relevant to irregular attendance than fear related to specific treatment procedures. In this sense, anticipatory DA may be particularly important at the stage of entry into care. This is in concordance with the finding that, among those who were afraid of visiting a dentist, the mean MDAS for anticipatory DA was higher than that for treatment-related DA [36]. Most studies of the association between DA and visiting oral health care are cross-sectional [4,7,10,11,12,16,22,23,24,25,26,27,28,29,40], and it is not possible to determine whether DA causes non-habitual use or vice versa. However, according to a longitudinal national study among adults in Finland, DA led to non-habitual use of services rather than vice versa [6], and similar results were found in a longitudinal cohort study in New Zealand [8].

In this study, men, those belonging to younger age groups, and those with lower education were also more likely to use oral health services non-habitually. Similarly, in a recent longitudinal study among adult Danes, non-users were younger, male, had lower income, lower education, and lower socioeconomic position [52]. However, in all studies, the association between younger age groups and non-habitual use of oral health care has not been found [53]. Also, in this study, the prevalence of non-habitual use of oral health care among 70+-year-olds was almost as high as among 20–39-year-olds.

This study has both strengths and limitations. In this Healthy Finland Survey 2023, a stratified, clustered random sample was used to ensure generalizability of the results to represent the adult population in the country. Inverse probability weights were used in the analyses to correct the population-level estimates and to reduce bias due to non-participation. The 2023 survey provides accurate information on changes over time because the study protocol has remained identical to previous population surveys in 2000 and 2011. Due to the cross-sectional nature of the 2023 study, causal interpretations are not possible. The smaller sample size in 2023 might have contributed to the wide confidence intervals. The validated MDAS questionnaire was used to measure DA, which was asked separately from oral health examinations, as those with high DA are less likely to participate in them. However, some of those with DA may also have avoided health examinations, potentially causing selection bias. Although people with DA avoid dental visits, they do not appear to be appreciably under-represented in oral epidemiological surveys [54]. Assessing DA in connection with health examinations might have had some effect, but might not have led to a considerable underestimation of the prevalence of DA or attenuation of the association between DA and habitual use of oral health care. In addition to the MDAS, DA was also assessed using a single validated question. This question allowed comparison of DA with previous national studies from 2000 and 2011, which did not include the MDAS; this omission represents a limitation of those studies. In this study, like in many other studies [55,56], non-participants were younger, less educated, and more often men and immigrants or their descendants. Participation rates in population studies have declined over the years [57]. These may limit the generalizability and comparability of the results, which are also limitations of this study. However, survey weights could be used to attenuate bias caused by non-participation.

In conclusion, in the Finnish national studies, among women, the prevalence of high DA decreased between 2000 and 2011, but the trend attenuated between 2011 and 2023. Among men, high DA decreased between 2000 and 2023. Overall, anticipatory and treatment-related DA acted as an independent barrier to the habitual use of oral health care. While the entire Finnish population is entitled to use public oral health care, barriers to use still exist. DA as an independent barrier can prevent habitual oral health care utilization, potentially leading to poor oral health. Thus, DA should be measured and attended to in oral health care.

## Figures and Tables

**Table 1:** Prevalence (%) with 95% confidence intervals (95% CI) of high dental anxiety from a single question in 2000, 2011, and 2023 by sex and age group.

	% (95% CI)								
Age Group	2000 † (n = 8081)			2011 † (n = 4091)			2023 ‡ (n = 1770)		
	All	Men	Women	All	Men	Women	All	Men	Women
20–29	10.0 (8.4;11.8)	7.4 (5.6;9.6)	12.7 (10.3;15.6)	4.3 (1.3;13.1)	0	11.4 (3.6;30.6)	3.8 (2.0;7.0)	3.0 (1.1;8.0)	4.4 (1.9;9.7)
30–39	11.8 (10.3;13.4)	7.0 (5.4;9.1)	16.7 (14.4;19.4)	8.5 (6.3;11.4)	5.2 (3.0;9.1)	11.5 (8.1;16.0)	8.2 (4.4;14.6)	3.7 (1.5;8.6)	12.1 (5.6;24.4)
40–49	12.1 (10.6;13.7)	9.3 (7.4;11.5)	14.9 (12.8;17.3)	7.6 (6.1;9.5)	5.2 (3.3;8.1)	9.7 (7.4;12.7)	9.0 (5.4;14.6)	5.5 (2.7;10.9)	13.1 (7.8;21.3)
50–59	8.9 (7.4;10.5)	5.2 (3.8;7.2)	12.5 (10.3;15.1)	7.0 (5.5;9.0)	4.6 (2.9;7.2)	9.2 (7.0;12.0)	6.5 (4,1;10.1)	2.6 (1.0;6.9)	10.1 (5.7;17.1)
60–69	8.2 (6.7;10.0)	5.8 (4.0;8.4)	10.2 (7.9;13.0)	5.6 (4.2;7.4)	4.5 (2.7;7.4)	6.7 (4.8;9.2)	4.2 (2.4;7.0)	1.1 (0.3;4.3)	7.0 (4.0;11.9)
70+	6.1 (4.6;8.0)	4.2 (2.6;6.7)	7.2 (5.3;9.6)	2.7 (1.7;4.2)	2.1 (0.9;4.6)	3.1 (1.9;5.2)	1.5 (0.7;3.1)	0.5 (0.1;3.3)	2.3 (1.0;5.2)

† follow-up survey for those aged 30+ at 2000, ‡ separate cross-sectional sample.

**Table 2:** Mean, standard error (SE), median, and interquartile range (IQR) of dental anxiety according to Modified Dental Anxiety Scale (MDAS) total and anticipatory and treatment-related subscales by sex and age group (n = 1739).

Age Group	MDAS Sum			MDAS Anticipatory Dental Anxiety					MDAS Treatment-Related Dental Anxiety			
	n	Mean (SE)	Median (95% CI)	IQR	n	Mean (SE)	Median (95% CI)	IQR	n	mean (SE)	Median (95% CI)	IQR
Women												
20–29	133	11.0 (0.8)	9.1 (7.6;10.6)	7.4	134	3.8 (0.3)	2.7 (2.3;3.1)	2.6	133	7.2 (0.6)	5.8 (5.0;6.6)	4.2
30–39	176	12.4 (0.3)	10.8 (10.2;11.4)	6.4	177	4.4 (0.1)	3.5 (2.8;4.3)	3.1	177	8.0 (0.2)	7.1 (6.5;7.8)	4.2
40–49	145	10.9 (0.4)	9.5 (8.5;10.4)	5.3	147	3.8 (0.2)	3.1 (2.4;3.9)	1.9	145	7.1 (0.2)	6.2 (5.8;6.6)	3.5
50–59	173	9.7 (0.3)	7.8 (7.3;8.4)	4.8	177	3.4 (0.2)	2.2 (1.7;2.6)	1.6	173	6.3 (0.2)	5.2 (4.8;5.6)	3.2
60–69	179	9.3 (0.3)	7.5 (6.9;8.0)	4.4	181	3.3 (0.1)	2.0 (1.7;2.3)	1.7	179	5.9 (0.2)	4.9 (4.6;5.3)	2.6
70+	183	7.6 (0.2)	6.5 (6.0;7.0)	3.8	191	2.8 (0.1)	2.0 (1.7;2.2)	1.2	184	4.9 (0.1)	4.0 (3.8;4.2)	2.5
Total	989	10.1 (0.1)	8.4 (8.1;8.8)	5.8	1007	3.6 (0.1)	2.5 (2.4;2.6)	1.8	991	6.5 (0.1)	5.4 (5.2;5.6)	3.7
Men												
20–29	81	8.7 (0.5)	7.3 (6.2;8.4)	5.7	83	3.1 (0.2)	2.0 (1.2;2.7)	1.6	81	5.6. (0.3)	4.9 (3.8;6.1)	3.7
30–39	142	9.9 (0.4)	8.7 (7.6;9.8)	5.5	143	3.6 (0.2)	2.7 (2.2;3.2)	1.9	142	6.3 (0.2)	5.3 (4.8;5.8)	3.1
40–49	122	8.9 (0.3)	7.0 (6.3;7.7)	5.0	122	3.2 (0.1)	2.0 (1.7;2.3)	1.5	122	5.7 (0.2)	4.6 (3.9;5.3)	3.4
50–59	130	8.5 (0.5)	7.3 (5.6;9.0)	4.1	132	2.9 (0.2)	2.0 (1.4:2.6)	1.3	131	5.5 (0.4)	4.7 (3.9;5.5)	2.7
60–69	139	7.9 (0.2)	6.6 (6.3;7.0)	4.6	141	2.8 (0.1)	2.0 (1.7;2.3)	1.1	139	5.1 (0.1)	4.4 (3.9;4.9)	2.8
70+	136	6.5 (0.2)	5.1 (4.9;5.3)	1.8	141	2.4 (0.1)	2.0 (1.3;2.7)	0	137	4.2 (0.1)	3.0 (2.8;3.3)	1.6
Total	750	8.4 (0.1)	6.9 (6.4;7.3)	4.8	762	3.0 (0.1)	2.0 (1.8;2.2)	1.2	752	5.4 (0.1)	4.5 (4.2;4.8)	3.0

## Data Availability

Data are not available due to privacy and ethical restrictions, in accordance with the EU GDPR and local legislation. Information regarding the requirements and procedures to request data access can be found at https://thl.fi/en/our-services/permit-and-data-requests (accessed 15 May 2026).

## Abbreviations

The following abbreviations are used in this manuscript:

Dental fear and anxiety	DA
Modified Dental Anxiety Scale	MDAS
